# Thoracoscopic oesophageal atresia/tracheo-oesophageal fistula (OA/TOF) repair is associated with a higher stricture rate: a single institution’s experience

**DOI:** 10.1007/s00383-020-04829-3

**Published:** 2021-02-07

**Authors:** H. Thakkar, D. M. Mullassery, S. Giuliani, S. Blackburn, K. Cross, J. Curry, Paolo De Coppi

**Affiliations:** 1 Department of Specialist Neonatal and Paediatric Surgery, Great Ormond Street Hospital, London, UK; 2 Stem Cells and Regenerative Medicine Section, Department of Paediatric Surgery, UCL Great Ormond Street Institute of Child Health, 30 Guilford Street, Holborn, London, WC1N 1EH UK

**Keywords:** Oesophageal atresia, Thoracoscopic, Minimal access surgery, Stricture

## Abstract

**Purpose:**

Thoracoscopic OA/TOF repair was first described in 1999. Currently, less than 10% of surgeons routinely employ minimally access surgery. Our primary aim was to review our immediate-, early- and long-term outcomes with this technique compared with the open approach.

**Methods:**

A retrospective review of all patients undergoing primary OA/TOF (Type C) repair at our institution from 2009 was conducted. Outcome measures included length of surgery, conversion rate from thoracoscopy, early complications such as anastomotic leak and post-operative complications such as anastomotic strictures needing dilatations. Fisher’s exact and Kruskal–Wallis tests were used for statistical analysis.

**Results:**

95 patients in total underwent OA/TOF repair during the study period of which 61 (64%) were completed via an open approach. 34 were attempted thoracoscopically of which 11 (33%) were converted. There was only one clinically significant anastomotic leak in our series that took place in the thoracoscopic group. We identified a significantly higher stricture rate in our thoracoscopic cohort (72%) versus open surgery (43%, *P* < 0.05). However, the median number of dilations (3) performed was not significantly different between the groups. There was one recurrent fistula in the thoracoscopic converted to open group. Our median follow-up was 60 months across the groups.

**Conclusion:**

In our experience, the clinically significant leak rate for both open and thoracoscopic repair as well as recurrent fistula is much lower than has been reported in the literature. We do not routinely perform contrast studies and are, thus, reporting clinically significant leaks only. The use of post-operative neck flexion, ventilation and paralysis is likely to be protective towards a leak. Thoracoscopic OA/TOF repair is associated with a higher stricture rate compared with open surgery; however, these strictures respond to a similar number of dilatations and are no more refractory. Larger, multicentre studies may be useful to investigate these finding further.

## Introduction

Oesophageal atresia with or without tracheo-oesophageal fistula (OA/TOF) is often considered one of the most technically challenging operations in neonatal surgery. Up until the turn of the century, the repair of this condition had only been performed via a thoracotomy. In 1999, Rothenberg et al. performed the first thoracoscopic repair of an infant with OA/TOF [1]. The main advantages of this approach have been considered the avoidance of a thoracotomy with the advantage of better cosmesis, less pain and reduced musculoskeletal morbidity such as scoliosis [2]. Parents and their family are also keen on minimally invasive approach and smaller scars [3] however in a survey of nearly 180 senior surgeons across Europe, only 6% preferred thoracoscopy as their favoured approach [4].

We have previously reported our institutional experience with this condition spanning nearly four decades [5–7] but have not previously compared our outcomes between the two approaches. We sought to review this in the last decade with a focus on our early and late complications.

## Methods

A decade-long (2009–till date) retrospective review of all patients undergoing primary OA/TOF (Type C) repair at our institution was conducted. Patients were divided into three groups for analysis: open, thoracoscopic converted to open and thoracoscopic. Patients who did not achieve an anastomosis at the initial attempt were excluded. Patients were identified through a prospectively maintained neonatal database and the trust audit department.

Thoracoscopic OA/TOF repair was performed using a 3-mm set with the infant in the semi-prone/lateral position. Insufflation pressures of 6–8 mmHg were used to create the pneumothorax. Open repair was performed via thoracotomy with an extra-pleural dissection. Chest drains were inserted at the end of most thoracoscopic procedures. Post-operatively, a 5-day period of paralysis, ventilation and neck flexion (PVF) was employed in patients with a tight oesophageal anastomosis. This decision was at the discretion of the individual surgeon performing the repair. We do not routinely perform a contrast study prior to commencing oral feeds.

Our outcome measures included conversion to open, length of surgery and immediate/early complications (< 4 weeks). Longer-term data on anastomotic strictures needing dilatations and fundoplication rates were also collected. Any patient with symptoms of stricture confirmed on a contrast study underwent dilatation performed in the interventional radiology department.

Fisher’s exact and the Kruskal–Wallis tests were used for statistical analysis with *P* < 0.05 considered to be significant.

## Results

95 patients in total underwent OA/TOF repair during the study period of which 61 (64%) were completed via an open approach. 34 (36%) were attempted via a thoracoscopic approach of which 22 were completed thoracoscopically and 11 converted to open. The median gestational age across these groups was the same. However, the thoracoscopic cases were significantly heavier and tended to have fewer cardiac co-morbidities (Table 1).

**Table 1:** Patient demographics as per the 3 cohorts in the study

	Open *n* = 61	Thoracoscopic *n* = 23	Thoracoscopic converted to open *n* = 11	Significance
Median gestational age (weeks, range)	37 (23–41)	38 (35–42)	38 (32–41)	NS
Median birth weight (g, range)	2287 (680–3570)	3030 (1790–4220)	3140 (1400–3800)	*P* < 0.05
Cardiac co-morbidities (%)	36 (59%)	10 (43%)	2 (18%)	*P* < 0.05

Of the 11 cases that were converted, the reasons for this were as follows; bleeding *n *= 1, right aortic arch with poor visualisation *n* = 1, small upper pouch with difficulties in mobilisation *n* = 5, difficulties in ventilation *n* = 1 and poor visualisation *n* = 3.

The operative data for the patients are as summarised in Table 2. The median anaesthetic time was significantly longer in the thoracoscopic cohorts as compared with open surgery. There was no significant difference when comparing the median time to extubation between the groups.

**Table 2:** Operative data as per the 3 cohorts in the study

	Open *n* = 61	Thoracoscopic *n* = 23	Thoracoscopic converted to open *n* = 11	Significance
Median anaesthetic time (minutes, range)	172.5 (90–510)	245 (165–420)	210 (145–350)	*P* < 0.05
Post-operative paralysis, ventilation and neck flexion (%)	44 (72%)	15 (65%)	9 (81%)	NS
Median time to extubation (days, range)	6.5 (1–28)	4 (1–8)	6 (1–15)	NS

We divided our complications into early as defined by those occurring  < 7 days post-operatively and longer term in keeping with outcomes that are routinely reported in the literature. One patient in the thoracoscopic group developed a clinically apparent anastomotic leak at post-operative day 4. This patient underwent a thoracoscopic re-exploration and repair of the leak (Tables 3, 4).

**Table 3:** Early complications (< 7 days) as per the three cohorts in the study

	Open *n* = 61	Thoracoscopic *n* = 23	Thoracoscopic converted to open *n* = 11	Significance
Chest infection (including aspiration)	4 (6.5%)	1 (4.3%)	2 (18%)	NS
Pneumothorax (managed conservatively without a chest drain)	4 (6.5%)	1 (4.3%)	1 (9%)	NS
Pneumothorax (managed with a chest drain)	3 (4.9%)	1 (4.3%)	1 (9%)	NS
Chylothorax	3 (4.9%)	0 (0%)	0 (0%)	NS
Vocal cord palsy	1 (1.6%)	0 (0%)	0 (0%)	NS
Respiratory arrest	1 (1.6%)	0 (0%)	0 (0%)	NS
Cardiac arrest (successful CPR)	1 (1.6%)	0 (0%)	0 (0%)	NS
Necrotising enterocolitis (NEC) (medical management)	1 (1.6%)	0 (0%)	0 (0%)	NS
Necrotising enterocolitis (NEC) (surgical management)	1 (1.6%)	0 (0%)	0 (0%)	NS
Wound breakdown	1 (1.6%)	0 (0%)	0 (0%)	NS
Intra-operative bleeding requiring transfusion	0 (0%)	1 (4.3%)	0 (0%)	NS
Venous thrombosis	0 (0%)	0 (0%)	1 (9%)	NS
Anastomotic leak	0 (0%)	1 (4.3%)	0 (0%)	NS

**Table 4:** Longer-term complications as per the three cohorts in the study

	Open *n* = 61	Thoracoscopic *n * = 23	Thoracoscopic converted to open *n* = 11	Significance
Symptomatic stricture needing dilatations (%)	26 (43%)	17 (71%)	8 (73%)	*P* < 0.05
Median number of dilatations (frequency, range)	3 (1–10)	3 (1–6)	4.5 (1–10)	NS
Recurrent fistula (%)	0 (0%)	0 (0%)	1 (9%)	NS
Fundoplication rate (%)	4 (6.5%)	1 (4%)	1 (9%)	NS
Median follow-up (months, range)	55 (2–111)	60 (2–120)	60 (3–120)	NS

There was a significantly higher symptomatic stricture rate in our thoracoscopic cohort as compared with those who underwent open surgery. However, the median number of dilatations was similar between the groups. There was one recurrent fistula identified in a patient who had thoracoscopic converted to open surgery.

There were 8 (13%) deaths in the open group. 6 patients died from their associated complex cardiac disease post-operatively. 1 patient suffered a fatal cardiac arrest in the intensive care following a pneumonia. 1 patient died from NEC. In the thoracoscopic group, there was also 1 (4%) death related to NEC. There were no deaths in the thoracoscopic converted to open group.

## Discussion

The minimal access (MAS) approach in neonatal surgery is increasingly being employed as a means of reducing operative morbidity. In several index conditions, this approach is now considered routine. However, despite first being described two decades ago, thoracoscopic OA/TOF repair is routinely performed in only a handful of surgical centres [4].

As with any operative approach, it is important to have selection criteria that will increase the likelihood of a successful outcome with minimal complications. In the context of neonatal MAS OA/TOF repair, several criteria have been proposed in the literature to optimise patient selection [8, 9]. This includes newborns that are stable from a cardiorespiratory perspective, no significant cardiac anomalies and are generally over 2 kg. In our series, the median birth weight of infants undergoing thoracoscopic surgery was over 3 kg compared with 2.3 kg in the open group. Our institution has a team of paediatric cardiac anaesthetists and thereby in our minimal access group, several patients with cardiac anomalies were still offered this approach as long as it was deemed safe from their perspective.

We were the first to describe that thoracoscopy can lead to hypercapnia and acidosis if high insufflation pressures are used [10]. However, we and others have demonstrated that this problem can be overcome by minimising the pressure required to maintain the pneumothorax [11]. Our median operative time in the thoracoscopic approach was longer than open repair. However, conversion to open surgery did not significantly elongate the operative time when compared to thoracoscopic repair. This was largely due to early decision making when faced with either not being able to progress in the procedure or inability to tolerate thoracoscopy.

The success of an oesophageal anastomosis is dependent on many factors but where possible tension on the repair in the post-operative period should be avoided. MacKinlay and Burtles first described in 1987 the use of elective paralysis, ventilation and neck flexion (PVF) in patients with wide-gap OA as a means of reducing post-operative complications [12]. The authors postulated that neck flexion would reduce the tension on the anastomosis through caudal movement of the upper pouch, whilst paralysis would reduce traction on the lower pouch that was attached intimately to the right crux of the diaphragm. This hypothesis was further tested and proven in porcine studies by Beasley et al. [13].

Most recently,  O’Connell JS et al. [14] have conducted a systematic review and meta-analysis on the use of this technique. Only three suitable studies could be included in the final analysis; however, the overall conclusion from the authors stated the use of PVF significantly reduced the incidence of anastomotic leak. The authors firmly believe the amelioration of the tension coupled with no active swallowing is protective of an anastomotic leak and continue to adopt this technique in the face of tension between the oesophageal ends. In our three cohorts of patients, more than 2/3 of all patients in each group received this technique.

Risk factors for anastomotic leakage include poor technique, ischaemia and severe tension. More than 70% of respondents in a survey of European surgeons reported routinely performing an oral contrast study post-operatively to check for a leak [4]. This has not been the practice in our institution. The incidence of post-operative leak has been widely reported to be between 10 and 20%. However, this figure is attained from several groups that do routinely perform this study [2]. In our series, the leak rate was significantly lower with only one clinically identifiable leak which occurred in the thoracoscopic group. The leak was identified early in this patient who had a successful thoracoscopic repair. Thoracoscopic repair of an early leak has also been described in the literature [15] and similar to the experience reported by the authors, in our case this patient did not have further identifiable leak. In summary, our clinically relevant leak rate is lower than that reported in the literature at least partly due to our policy of not routinely “screening” patients for it and the use of PVF as described above.

The long-term morbidity from oesophageal atresia is accounted for by oesophageal dysmotility, gastro-oesophageal reflux, respiratory complications and anastomotic strictures. A recent review of outcomes spanning across the last eight decades has quoted the rate of strictures to be between 20 and 40% [16]. In 2014, the UK BAPS-CASS group reported a 36% stricture rate prior to one-year post-anastomosis in 76 surviving infants [17].

In our institution, symptomatic oesophageal strictures are dilated in the interventional radiology department. Balloon dilatation of strictures has been shown to be both safe and associated with a low perforation rate [18]. The stricture rate reported in our cohort of patients undergoing thoracoscopic surgery (71%) was significantly higher compared with our open group (43%).

The risk factors for anastomotic stricture post-repair include anastomotic leak, ischaemia, tension as well as the use of trans-anastomotic feeding tubes (TAT) [19]. As discussed above, our clinically significant leak rate was much lower than that reported in the literature. Although we do not perform routine oral contrast studies, we did not identify any other clinical signs that may signify a leak except in one case as described above. Thoracoscopic oesophageal atresia repair is technically very challenging with a learning curve demonstrated by several groups [20, 21]. The handling of the tissues can be more traumatic in this cohort of patients compared with open surgery which may have contributed to more scarring and stricturing. Some authors have reported between 10 and 20 cases to attain a stable learning curve which emphasis the need to concentrate surgical experience within individuals within institutions [9, 21].

In all our patients, we place a trans-anastomotic feeding tube (TAT) as a means of establishing early enteral feeding, administration of medications and facilitating gastric drainage. As observed by Wang et al., a higher stricture rate in patients with a TAT tube [19] as compared to those without has been reported. On balance, we have nonetheless felt that the advantages of using a TAT outweigh any potential harm which is currently only supported by poor evidence in the literature. This is keeping with recent consensus conference published by ERNICA which recommends the use of a TAT tube [9].

Yang et al. [2] conducted a meta-analysis and systematic review comparing the outcomes between open and thoracoscopic surgery. They showed no significant difference in stricture rates. Similarly, a Canadian group of researchers also reported no difference in outcomes between the two groups [22]. In another series of patients from Japan, again no difference has been identified in their cohort of patients [23]. However, what it is clear from this study and several other publications on the long-term stricture rate is what we would consider as the definition of a stricture. In the study by Yamoto et al. [23], all patients post-operatively received a balloon dilatation at 4 weeks (6–8 mm) and the authors defined a stricture as one that required at least two dilatations. In our centre, symptomatic patients were investigated with a contrast study followed by a dilatation if proven to have a stricture. The median number of dilatations identified between our groups was similar and therefore, although we are experiencing a higher stricture rate in the thoracoscopic group, these strictures are not more recalcitrant. Vergouwe et al. [24] identified anastomotic leak and the need for a dilatation within the first month after surgery as significant risk factors for developing a refractory stricture. We have only identified one early clinical leak in our thoracoscopic group with no patients requiring a dilatation within 28 days of surgery.

In conclusion, in our experience, the clinically significant leak rate for both open and thoracoscopic repair as well as recurrent fistula is much lower than has been previously reported in the literature. The use of post-operative paralysis, ventilation and neck flexion we believe is protective towards a leak. Thoracoscopic repair is associated with a higher stricture rate compared with open surgery; however, these strictures respond to a similar number of dilatations and are no more refractory. Larger, multicentre studies may be useful to investigate these findings further.
